# Arthropod Venom Components and Their Potential Usage

**DOI:** 10.3390/toxins12020082

**Published:** 2020-01-25

**Authors:** Gandhi Rádis-Baptista, Katsuhiro Konno

**Affiliations:** 1 Laboratory of Biochemistry and Biotechnology, Institute for Marine Sciences, Federal University of Ceara, Fortaleza CE 60165-081, Brazil; 2 Institute of Natural Medicine, University of Toyama, 2630 Sugitani, Toyama, Toyama 930-0194, Japan

Arthropods comprise a predominant and well-succeeded phylum of the animal kingdom that evolved and diversified in millions of species grouped in four subphyla, namely, Chelicerata (arachnids), Crustacea, Myriapoda (centipedes), and Hexapoda (insects). It is agreed that the success of the arthropods’ flourishment and evolutionary story are in great part due to the diversification of venom apparatus and venom usage [1,2]. Thousands of arthropod species, ranging from arachnids (spiders and scorpions) to hymenopterans (ants, bees, and wasps) and myriapods (centipedes), are venomous and utilize their venoms for chemical ecological warfare that includes individual and colonial defense, predation, and paralysis of coexistent species to nourish their brood. Despite arthropods’ venoms are invariably harmful to humans, and some may cause serious injuries, e.g., those from scorpions, spiders, and wasps, they are potentially useful molecular scalpels to dissect and modulate cellular processes and, consequently, they can be converted into biopharmaceuticals and biotools. In this respect, arthropod venoms have attracted the attention of toxin researchers for years, seeking to characterize biologically active compounds of these rich venom sources. Especially in the last decades, venom component analysis has progressed more than ever because of the great advances of analytical techniques; in particular, mass spectrometry and next-generation deep (DNA and RNA) sequencing. As such, proteomic and peptidomic analyses utilizing LC–MS, as well as transcriptomics (alone or in combination with proteomics), have made it possible to fully analyze venom components, revealing a variety of novel peptide and protein toxin sequences and scaffolds. These are potentially useful as pharmacological research tools and for the development of highly selective peptide ligands and therapeutic leads. Moreover, because of their specificity for numerous ion-channel subtypes, including voltage- and ligand-gated ion channels, arthropod neurotoxins have been investigated to dissect and treat neurodegenerative diseases and control epileptic syndromes. This Special Issue collects information on such progress. 

Considering the natural history of the evolutionary success of arthropods based on the molecular arsenal contained in their venom, a study reported here by Justin Schmidt explores and correlates the pain and lethality induced by hundreds of insect stings, pointing the direction to screen pharmacologically active venom components of pharmaceutical interest [3]. To dissect venom cocktails, particularly when limited amounts of crude venom are available from tiny animals, as in the case of most arthropod species, omics technologies have demonstrated to be an essential collection of robust strategies. Indeed, transcriptome and proteome, alone or in combination with functional analysis, has been applied to disclose and resolve the toxin peptide complexity of the venom, as described from the highly venomous Mexican scorpion *Centruroides limpidus* [4], the predatory giant ant *Dinoponera quadriceps* [5], and the predatory ant *Odontomachus monticola* [6]. In a later study published in this special issue, the authors also investigated the components of the *O. monticola* venom sac, besides the crude venom. Apart of numerous structural and functional classes of polypeptides found in a given venom proteome and peptidome, short membrane active peptides with or without definitive characterized antimicrobial activity have also been found in the venom of these species of ant and scorpion, like in other arthropods. The structural and molecular characterization of antimicrobial peptides are the focus of four articles: the antimicrobial and antibiofilm effects of peptides agelaia-MPI, polybia-MPII, polydim-I from the venom of social wasps, and the peptides Con10 and NDBP5.8 from scorpion venom against multidrug-resistant *Acinetobacter baumannii*, investigated and reported by das Neves and colleagues [7]; a detailed study on the chemical, biological, and biophysical properties of antimicrobial alpha-helical peptides from solitary wasp venoms, presented by dos Santos Cabrera and collaborators [8]; the formulation of a new topical eye drop containing a synthetic peptide designed from a spider *A. lycosa erithrognata* venom toxin, LyeTxI-b, that is effective in treating bacterial keratitis caused by drug-resistant Staphylococcus aureus, reported by Nunes da Silva et al. [9]; the arthropod venoms as a source of antimicrobial peptides that kill diverse life-threating parasites, reviewed by Sabia-Junio et al. [10]. In addition to antimicrobial and antiparasitic peptides from arthropod venom, low molecular weight compounds are also shown to be active against a broad spectrum of microbes. For instance, the anti-biofilm effect of alkaloids (solenopsins) isolated from the venom of the fire ants *Solenopsis invicta* was evaluated by de Carvalho and colleagues [11]. Cantharidin, a toxic monoterpene from the hemolymph of the blister beetles *Berberomeloe majalis* (Coleoptera: Meloidae), was demonstrated by Whitman and coworkers to display an important effect against distinct class of parasites [12].

One of the most studied animal venoms, bee venom, still has many interesting aspects to be discovered and explored. Crude venom and isolated components were reexamined in a review dealing with the potential therapeutic applications of bee venom to treat skin diseases [13], and in three different research articles dealing with bee venom peptides, melittin and tertiapin, from the view of immunology, molecular neurobiology and physiology. Indeed, Lubawy and collaborators studied the immunotropic and cardiotropic effects of melittin on the physiology of beetle *Tenebrio molitor* [14], while Choi and coworkers investigated the use of melittin as an analgesic to treat peripheral neuropathy caused by oxaliplatin (an anticancer drug), demonstrating the molecular basis of this particular melittin effect, which was mediated by the activation of the spinal α1- and α2-adrenergic receptors [15]. In another work, the Kir channel subtypes of the small hive beetle *Aethina tumida* were identified by Doupnik [16] as molecular targets of the bee venom peptide tertiapin, based on structure-guided virtual screening methods. 

Neural receptors on excitable tissues, particularly ion channels, are a sort of preferential targets for arthropod venom components, notably from spider and wasps. Dongol and coworkers reviewed the structural determinants of diverse spider knottins (inhibitor cystine knot toxins) that influence voltage-gated sodium (Nav) channel activity on neuronal signaling, their role in the modulation of pain, and as a platform to develop analgesics [17]. In the same line, Chaves-Moreira and collaborators explored the potential of distinct structural and functional classes of toxins from brown spider (*Loxoceles*) to be developed into therapeutics [18]. The purification and preparation of fully bioactive peptide toxins, particularly folded and constrained by disulfide bonds, are critical for functional analysis and development as biopharmaceuticals. Nicolas and colleagues synthesized and characterized in a structural and functional basis a spider peptide toxin, phlotoxin-1, that was specifically selective to Nav channel and, consequently, useful to investigate the involvement of sodium channel in pain and analgesia [19]. Acid-sensing ion channels (ASICs) comprise another family of proton-gated ion channel expressed in the nervous system and with multiples roles in organism physiology and neurological diseases. Hernández and colleagues reported the effect of two peptides purified from the solitary wasp *Sphex argentatus*, Sa12b and Sh5b, on ASIC currents in rat dorsal root ganglion neuron, contributing with the first discovery of a wasp peptide toxin that acts on such a kind of ion channel [20]. The preparation of toxin with sizes exceeding those of peptides can be achieved by recombinant procedures instead of solid phase peptide synthesis chemistry. An example of this alternative in the present special issue is the production of recombinant hybrid toxin/immunogen. Taking phospholipase D from the spider *Loxoceles* as toxin moiety, a chimeric hybrid was produced by Calabria and colleagues to raise protective antibodies in *Loxoceles* antivenom therapy [21]. Last but not least, the use of arthropod toxins as bioinsecticide is continuously showed to be a promising application of this classes of animal venom. Yoshimoto and collaborators described the isolation and molecular characterization of insecticidal toxins from the venom of the North African scorpion, *Buthacus leptochelys* [22]. These new toxins were shown to be similar to scorpion α- and β-toxins and probably acted via sodium ion channels. 

Overall, the compilation of such special articles highlights the huge potential of the discovery of arthropod venom. The diversity of peptide scaffolds and structures found in the numerous species of arthropods are amenable to be developed into specific and selective ligands and biotools. These, apart from being useful in basic research, are usable for precise intervention and modulation of the physio-pathological processes of diseases such as neurological disorders, or even for pest control, such as in the preparation and use of environmentally friendly biopesticides. So far, the future is bright for the usage of selective arthropod peptides.
